# Targeting both IGF-1R and mTOR synergistically inhibits growth of renal cell carcinoma *in vitro*

**DOI:** 10.1186/1471-2407-13-170

**Published:** 2013-04-01

**Authors:** Thomas M Cardillo, Preeti Trisal, Roberto Arrojo, David M Goldenberg, Chien-Hsing Chang

**Affiliations:** 1 Immunomedics, Inc, 300 American Rd, Morris Plains, NJ 07950, USA; 2 IBC Pharmaceuticals, Inc, 300 American Rd, Morris Plains, NJ 07950, USA; 3 Center of Molecular Medicine and Immunology, Garden State Cancer Center, 300 American Rd, Morris Plains, NJ, 07950, USA

**Keywords:** Dock-and-Lock, Renal cell carcinoma, Insulin-like growth factor-I receptor, Hex-hR1, 1R-2b, mTOR inhibitors

## Abstract

**Background:**

Advanced or metastatic renal cell carcinoma (RCC) has a poor prognosis, because it is relatively resistant to conventional chemotherapy or radiotherapy. Treatments with human interferon-α2b alone or in combination with mammalian target of rapamycin (mTOR) inhibitors have led to only a modest improvement in clinical outcome. One observation made with mTOR inhibitors is that carcinomas can overcome these inhibitory effects by activating the insulin-like growth factor-I (IGF-I) signaling pathway. Clinically, there is an association of IGF-I receptor (IGF-IR) expression in RCC and poor long-term patient survival. We have developed a humanized anti-IGF-IR monoclonal antibody, hR1, which binds to RCC, resulting in effective down-regulation of IGF-IR and moderate inhibition of cell proliferation *in vitro*. In this work, we evaluate the anti-tumor activity of two novel IGF-1R-targeting agents against renal cell carcinoma given alone or in combination with an mTOR inhibitor.

**Methods:**

hR1 was linked by the DOCK-AND-LOCK™ (DNL™) method to four Fabs of hR1, generating Hex-hR1, or to four molecules of interferon-α2b, generating 1R-2b. Eight human RCC cell lines were screened for IGF-1R expression and sensitivity to treatment with hR1 *in vitro*. Synergy with an mTOR inhibitor, temsirolimus, was tested in a cell line (ACHN) with low sensitivity to hR1.

**Results:**

Hex-hR1 induced the down-regulation of IGF-IR at 10-fold lower concentrations compared to the parental hR1. Sensitivity to growth inhibition mediated by hR1 and Hex-hR1 treatments correlated with IGF-1R expression (higher expression was more sensitive). The potency of 1R-2b to inhibit the *in vitro* growth of RCC was also demonstrated in two human cell lines, ACHN and 786-O, with EC_50_–values of 63 and 48 pM, respectively. When combined with temsirolimus, a synergistic growth-inhibition with hR1, Hex-hR1, and 1R-2b was observed in ACHN cells at concentrations as low as 10 nM for hR1, 1 nM for Hex-hR1, and 2.6 nM for 1R-2b.

**Conclusions:**

Both Hex-hR1 and 1R-2b proved to be more potent than parental hR1 in inhibiting growth of RCC *in vitro*. Synergy was achieved when each of the three hR1-based agents was combined with temsirolimus, suggesting a new approach for treating RCC.

## Background

In the United States, renal cell carcinoma (RCC) is the seventh and ninth most common form of cancer in men and women, respectively, and a recent report estimates that in 2012, 40,250 men and 24,520 women will be diagnosed with, and 13,570 will die of, this disease [1]. The therapeutic options for RCC have increased considerably since 2005, due to the availability of seven new agents [2,3] developed to interrupt the molecular pathways regulating tumor angiogenesis, cell proliferation, and survival. Treatments of metastatic RCC with these agents, which are inhibitors of vascular endothelial growth factor (VEGF) (bevacizumab), VEGF-receptors (VEGFR) (sorafenib, sunitinib, pazopanib, and axitinib), or mTOR (temsirolimus and everolimus), have a significantly improved survival, but remain palliative. Thus, a cure for metastatic RCC continues to be elusive, but is being pursued actively with various combination strategies. In this respect, it is noted that the time-honored, but not regulatory-approved, therapies with interferon-alpha (IFN-α) have had mixed results in RCC when used in combination with some of these agents. For example, IFN-α2b combined with sorafenib achieved an overall response rate of 33% in patients with metastatic disease [4]; IFN-α2b combined with temsirolimus was not as effective as temsirolimus alone [5]; and IFN-α combined with bevacizumab significantly increased progression-free survival and objective responses [6]. One major challenge with IFN-α therapy, either alone or in combination, is the frequency with which IFN-α needs to be administered (6 to 10 ×10^6^ U three times weekly). Another problem associated with IFN-α therapy was the adverse events, which include fatigue, fever, nausea, flu-like symptoms, and anorexia [4,6]. While pegylated IFN-α has allowed for less frequent dosing, many of the same toxic effects remain without appreciable improvement in patient outcome [7,8].

Inhibition of the mTOR kinase results in the reduction of regulatory proteins involved in the progression of cells from the G_1_ to S-phase of their growth cycle [9]. However, blocking mTOR activity with rapamycin (the prototype mTOR inhibitor) or rapamycin analogs inadvertently activates the Akt-signaling pathway [9] through an IGF-1R-dependent mechanism [10], which mitigates the anti-tumor effects of the mTOR inhibitors. Thus, the combination of an anti-IGF-IR antibody with mTOR inhibitors was shown to block the Akt-signaling pathway in rhabdomyosarcoma, breast, and prostate carcinomas, resulting in an additive increase in cell growth-inhibition [9,10].

IGF-1R-targeted therapy in RCC was implicated by an early finding of a greater than 10-fold reduction in tumor growth in mice bearing xenografts of human clear-cell RCC when administered with an antagonist to growth hormone-releasing hormone, which was attributed to a reduction of IGF-1 [11]. Analysis of RCC tissue specimens showed the expression of both IGF-1 and IGF-IR in clear-cell-RCC, papillary-RCC, and chromophobe RCC [12]. Overall, an association of IGF-1R expression and poor long-term patient survival was found, particularly among patients with high-grade tumors [13]. Mutations in the von Hippel-Lindau (VHL) gene have been linked to hereditary kidney cancer and in 70% of non-hereditary clear-cell-RCC [14]. It was shown that the wild type VHL encodes a 30-kDa protein that inhibits RCC metastasis and IGF-IR to form complexes with PKCδ, a protein kinase linked to cell proliferation and transformation [15].

We have developed a humanized anti-IGF-1R antibody, hR1, which binds to IGF-1R without blocking binding of IGF-1 or IGF-2 to the receptor, yet effectively causes receptor down-regulation, and inhibits cell proliferation, colony formation, and cell invasion in a variety of cancer types, including breast, prostate, cervical, pancreatic, and rhabdomyosarcoma [16]. Additionally, using the DOCK-AND–LOCK™ (DNL™) platform technology [17,18], a hexavalent form of hR1 (Hex-hR1) was engineered in which four hR1 Fabs were linked to hR1 IgG [16]. Hex-hR1 and hR1 were found to have similar activity, although Hex-hR1 was more effective at down-regulating IGF-1R. Importantly, both hR1 and Hex-hR1 were able to significantly inhibit the anchorage-independent growth of two different RCC lines in soft-agar assays. When both hR1 and Hex-hR1 were combined with rapamycin treatment of mice bearing a human rhabdomyosarcoma, significant tumor growth inhibition was achieved in comparison to either agent used alone [16].

This same DNL technology can be utilized to attach four molecules of IFN-α to hR1. It has already been demonstrated that by using this method with an anti-CD20 antibody, a significant improvement in therapeutic efficacy in mice bearing xenografts of human non-Hodgkin lymphoma is achieved when compared to either the parental antibody alone or peginterferon alfa-2a [19]. By attaching the IFN-α2b to an antibody that targets the tumor, the therapeutic window of IFN-α should improve by concentrating IFN-α at the tumor, while at the same time decreasing the amount in the blood and normal tissues, where its toxicity manifests.

The known association of mTOR and IGF-IR signaling pathways, along with the correlation in IGF-1 and IGF-IR expression patterns in RCC, provide an attractive rationale for a combination therapy. Moreover, with current IFN-α treatments and the added benefit already observed in a rhabdomyosarcoma tumor model with an anti-IGF-IR antibody enhancing the therapeutic effects of mTOR inhibitors or to specifically target IFN-α to a tumor, there is the potential to provide a new combination therapy for metastatic RCC. We report here that a screening of eight different human RCC cell lines reveals that all eight express IGF-1R at varying levels. Sensitivity to IGF-1 stimulation and growth-inhibitory effects of hR1 or Hex-hR1 are related to this expression. The hR1-IFN-α2b DNL product, 1R-2b, was found to have activity similar to recombinant human IFN-α and could inhibit RCC growth with EC_50_-values in the picomolar range. Importantly, there is a strong synergistic effect when hR1, Hex-hR1, or 1R-2b is combined with the rapamycin analog, temsirolimus.

## Methods

### Cell lines, antibodies, and reagents

All cell lines were purchased from American Type Culture Collection, except CAL-54 and RH-30, which were obtained from the Deutsche Sammlung von Mikroorganismen und Zellkulturen. Humanized antibodies, including hR1, hA20 (anti-CD20), hRS7 (anti-Trop-2), and h225 (anti-epidermal growth factor receptor (EGFR)), were provided by Immunomedics. Recombinant human IGF-1 and murine anti-human IGF-1R MAb (MAB391) were obtained from R&D Systems. Phospho-specific antibodies and other primary antibodies were acquired from Cell Signaling or Santa Cruz Biotechnology. Horseradish peroxidase (HRP)-conjugated secondary antibody and One Solution Cell Proliferation assay (MTS) were obtained from Promega. FITC-conjugated secondary antibodies were from Jackson ImmunoResearch Laboratories. PhosphoSafe Extraction Reagent and RIPA buffer used for cell lysis were obtained from EMD Biosciences and Cell Signaling, respectively. Cell culture media, supplements, and bovine transferrin (holo form) were purchased from Invitrogen. Temsirolimus (Wyeth) was purchased from Florida Infusion. Recombinant human Interferon-α2a (rhIFN-α2a, Millipore), peginterferon alfa-2b (Schering) and peginterferon alfa-2a (Hoffmann-La Roche) were purchased. Protein Assay Dye Reagent Concentrate was from Bio-Rad. All other chemicals were purchased from Sigma.

### Cell culture

RCC cell lines 769-P and 786-O were maintained in RPMI-1640 medium. For CAL-54, A-498, A-704 and ACHN, Eagle’s MEM medium was used, and for Caki-1 and Caki-2, McCoy’s 5a medium. All three media were supplemented with 10% heat-inactivated fetal bovine serum (FBS), 1% GlutaMax, 1% non-essential amino acids, and 1% sodium pyruvate. Cultures were maintained at 37°C in 5% CO_2_ and medium changed at least once weekly. Only cells with fewer than 50 passages were used for experiments.

### Generation of Hex-hR1 and 1R-2b by DNL

The preparation of Hex-hR1 has been described [16]. 1R-2b was prepared as described for 20-2b [19] by reacting C_H_3-AD2-hR1-IgG, instead of C_H_3-AD2-hA20-IgG, with IFN-α2b-DDD2. The molecular integrity and product purity of Hex-hR1 and 1R-2b were determined by size-exclusion high performance liquid chromatography (SE-HPLC) on a Beckman System Gold Model 116 with a BioSep-SEC-s3000 column (300 × 7.80 mm) of Phenomenex using 0.04 M PBS (pH 6.8) plus 1 mM EDTA as the mobile phase.

### Surface IGF-1R expression by flow cytometry

Each sample was prepared in duplicate to contain 2×10^5^ cells and 67 nM of a test antibody in a final volume of 200 μL. After incubation at 4°C for 45 min, samples were washed twice with PBS-1% BSA, followed by the addition of FITC-GAH IgG, (H + L), and a further incubation at 4°C for 45 min in the dark. Samples were washed twice with PBS-1% BSA, resuspended in 500 μL of PBS-buffered formalin, and analyzed on FACScan.

### Cell proliferation assay

All cell incubations were performed at 37°C in a humidified 5% CO_2_ incubator. Cells were detached with trypsin, washed three times with PBS to remove any trace of serum, and resuspended in a serum-free medium containing 10 μg/mL of bovine transferrin (SFM-Trf). Cells were seeded at 1×10^3^ cells/50 μL/well and incubated overnight. On the following day each test article in SFM-Trf was 5-fold serially diluted from 400 nM to 0.001 nM and 50 μL of each concentration were added in triplicate to the wells, such that the final concentrations of the test article ranged from 200 nM to 0.0005 nM. Untreated control cells received only 50 μL of SFM-Trf. After incubation for 1 h, designated wells received 100 μL of each test article at the same concentration in SFM-Trf containing 50 ng/mL of IGF-1. Plates were then incubated for a period of time as indicated and cell viability assessed using the MTS assay as per the manufacturer’s protocol. Growth inhibition was measured as a percent of growth relative to untreated cells using Microsoft Excel and Prism GraphPad Software (v4.03; Advanced Graphics Software, Inc.). Combinatorial Index (CI) was calculated by median effect analysis [20,21] to determine synergism (CI < 0.9), additivity (1.1 > CI > 0.9), or antagonism (CI >1.1).

### Immunoblot analysis

Unless otherwise stated, cells were starved in serum-free medium for 24 h, treated, and lysed at ice-cold temperature in a buffer as specified. Protein concentrations were determined by the Bio-Rad Protein Assay and samples (20 μg loaded in each lane) were separated on 4-20% Tris-Glycine gels, transferred to PDVF or nitrocellullose membranes, blocked with TBST buffer (50 mM Tris pH 8.0, 150 mM NaCl, 0.1% Tween 20) containing 5% nonfat milk, washed with TBST buffer, and incubated overnight at 4°C with primary antibodies. The membranes were then washed in TBST four times (once for 15 min and three more for 5 min each), incubated with HRP-conjugated secondary antibodies for 1 h at RT, washed in TBST buffer four times as described above, then detected with Super Signal West Dura Extended Duration Substrate (Thermo Scientific) according to the directions provided by the manufacturer. The immunoblot signals were visualized with a chemiluminescence system (Thermo Scientific). Digital images were processed by Carestream (Carestream Molecular Imaging).

### Down-regulation of IGF-IR

Cells were seeded at 1×10^6^ per well in a 6-well plate and cultured overnight for attachment. On the next day, the medium was replaced with fresh media containing a test article of interest at indicated concentrations and cells were further incubated as indicated. Treated cells were washed with cold PBS, scraped from the dishes, collected, and centrifuged at 4°C at 2,000 rpm for 5 min. Cells pellets were lysed for 10 min on ice in RIPA buffer or a buffer consisting of 25 mM Tris (pH 8), 150 mM NaCl, 1 mM EDTA, 1% Triton and 1 X Complete, EDTA-free Protease Inhibitor Cocktail (Roche Diagnostics). The lysates were clarified by centrifugation, assayed for protein concentration, and analyzed by immunoblotting.

### Detection of IGF-1R/IR hybrid

Cells grown in T150 flasks were washed twice with ice-cold PBS and scraped by adding ice-cold lysis buffer. Lysates were centrifuged for 15 min at 13,000 × g at 4°C. Supernatants were assayed for protein content using the BCA assay kit. Aliquots of 500 μg of total protein in equal amount of lysis buffer were pre-cleared with protein A beads (Cell Signal Technology) for 2 h. The pre-cleared lysate was incubated overnight at 4°C with anti-IGF1R antibody (MAB391,1:100, Santa Cruz Biotechnology). Samples were then incubated with 40 μL of protein A beads for 2 h. The beads were washed four times with lysis buffer and collected by centrifugation. Beads were resuspended in 2× sample buffer and boiled for 5 min. A 20-μL aliquot of the supernatant was subjected to gel electrophoresis and Western blotting with an anti-insulin receptor-β antibody (anti-IR-β) to detect heterodimers. As a control for the presence of IGF-1R in the immunoprecipitated samples, the blots were also probed with an anti-IGF-1Rβ antibody. IR-β levels were assessed by running 25 βg from the same lysates used for immunoprecipitation and probed with anti-IR-β antibody. Loading control was β-actin.

### Determination of IFN activity

Two different assays were utilized to determine the specific activity of 1R-2b. The first used the luciferase reporter gene assay (iLite kit; PBL InterferonSource) following manufacture’s instructions in which rhIFN-α2a was used as a standard for activity. A second measure of activity tested the ability of IFN-α2a to mediate the phosphorylation of STAT1, AKT or ERK1/2 in ACHN cells. Briefly, cells (5 × 10^5^ per well) were grown in 10% FBS medium in 6-well plates overnight for attachment. Medium was changed and interferon (rhIFN-α2a or 1R-2b) was added at the indicated concentrations. At indicated times, cell lysates were prepared and resolved by SDS-PAGE, transferred to nitrocellulose membranes and probed with appropriate anti-phospho antibodies (p-STAT1, p-ERK1/2, p-AKT) or anti-NUB1. Loading controls utilized antibodies to unphosphorylated proteins (STAT1, ERK1/2, AKT) or to β–actin (NUB1).

### Statistical analysis

Results are shown as means ± standard deviations. Statistical differences between two values were determined by Student’s *t*-test. A value of *P* < 0.05 was considered statistically significant.

## Results

### Characterization of Hex-hR1 and 1R-2b

**Figure 1:**
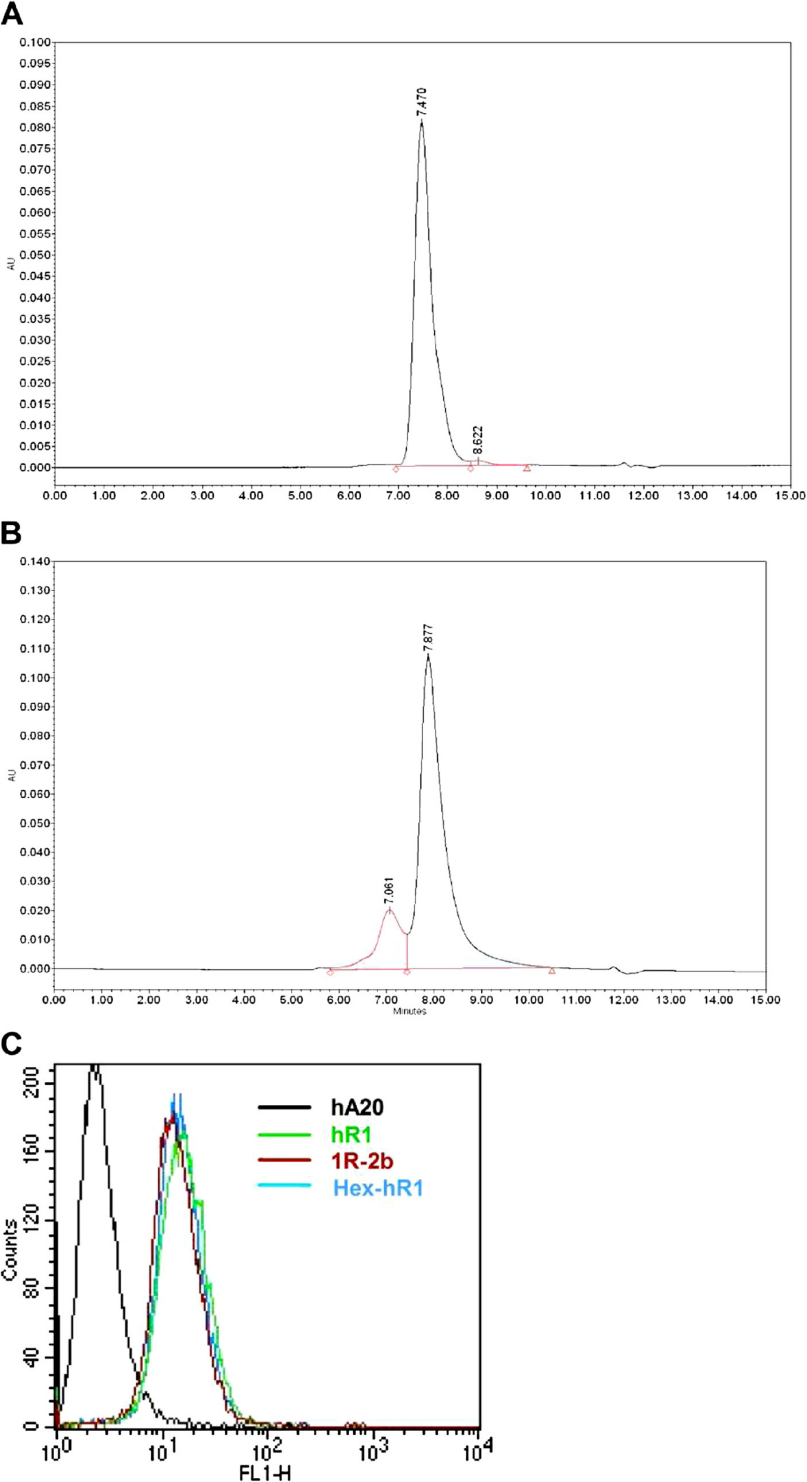
**Characterization of Hex-hR1 and 1R-2b. **A 50-μg sample of (**A**) Hex-hR1 or (**B**) 1R-2b was run on SE-HPLC as described in Materials and Methods. Histograms show UV absorbance of eluted material *versus *time. (**C**) Cell binding of hR1, Hex-hR1, and 1R-2b at equimolar concentrations on ACHN cells by FACS staining. Anti-CD20 antibody, hA20, served as negative control.

Hex-hR1 and 1R-2b were analyzed by SE-HPLC, showing a single peak at 7.47 min (Figure 1A) and a major peak at 7.88 min (Figure 1B), respectively. The smaller peak observed for 1R-2b at 7.06 min (16% area) is presumably a dimer of 1R-2b, due to the propensity of interferon to self-associate [19]. As shown in Figure 1C, both Hex-hR1 and 1R-2b bind to ACHN cells with no appreciable difference from the parental hR1.

### Down-regulation of IGF-1R

**Figure 2:**
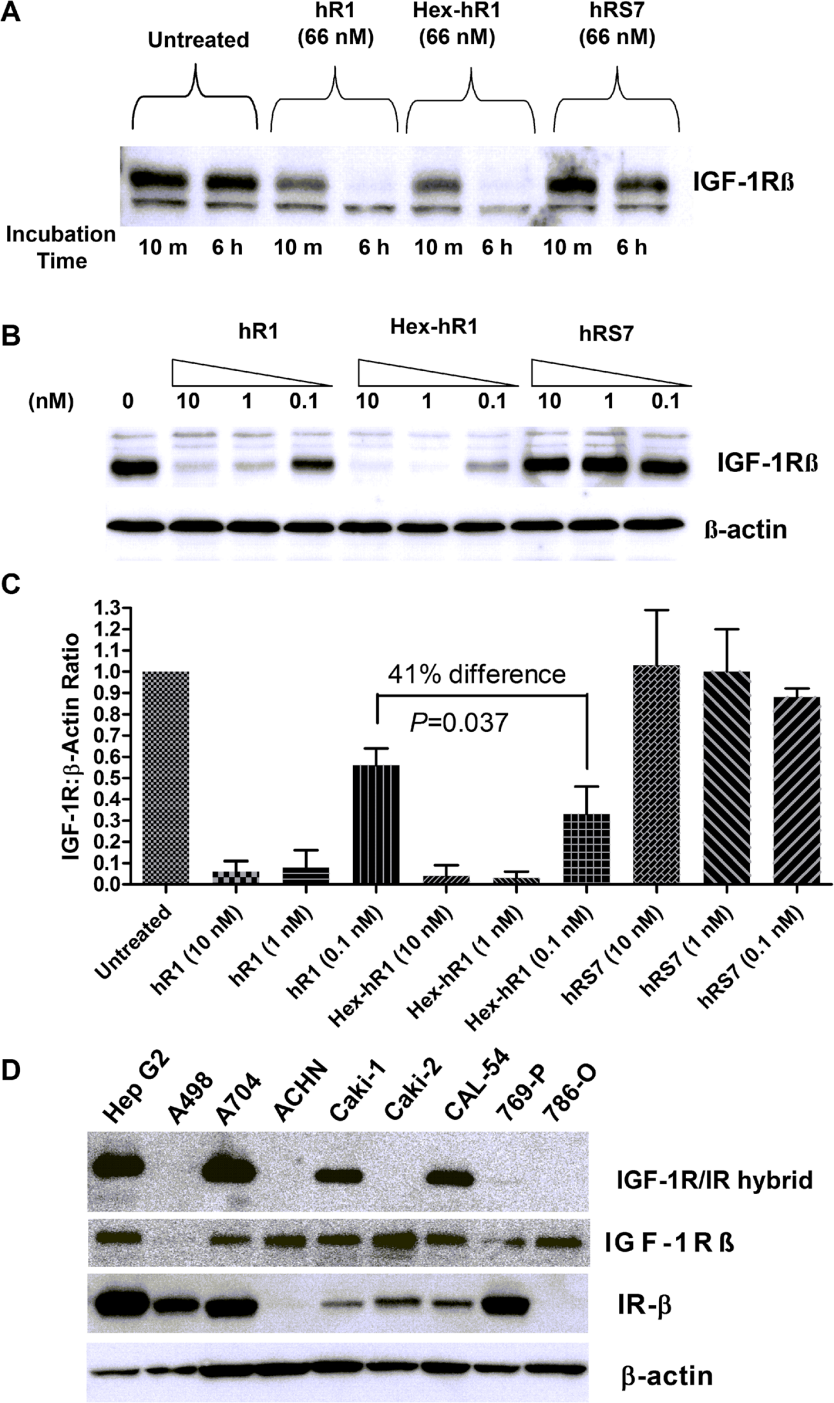
**IGF-1R expression. **(**A**) ACHN cells were plated overnight in 6-well plates as described in Materials and Methods. Cells were than exposed to hR1, Hex-hR1, or control hRS7 antibodies before being lysed and 20 μg protein from these lysates subjected to SDS-PAGE (4-20%) followed by blotting with an anti-IGF-1Rβ antibody. IGF-1R time-course down-regulation after exposure to constant amount of either hR1 or Hex-hR1. (**B**) Cells were exposed to indicated amounts of hR1, Hex-hR1, or hRS7 (anti-Trop-2 antibody) for 6 h before being lysed for Western blotting. β-actin served at the loading control. Blot shown is representative of three repeat experiments. (**C**) Ratio of IGF-1R to β-actin loading control normalized to untreated cells for the various doses of hR1, Hex-hR1 or control hRS7. Data are shown as mean ± standard deviation. Significance set at *P* < 0.05 for paired *t*-test of three experiments. (**D**) Indicated cells were lysed subjected to immunoprecipitation to determine IGF-1R/IR hybrid receptors, as described in Materials and Methods. A 20-μL aliquot from each immounoprecipitation preparation was subjected to gel eletrophoresis and Western blotting. Hep G2 served as the positive control cell line in these experiments [22]. Blot shown is representative of two repeat experiments. To confirm presence of IGF-1R in IP samples, IP blots were probed with anti-IGF-1R antibody. Additionally, cell lysates were subjected to Western analysis and probed with an anti-IRβ antibody to show relative levels of IR in the various cell lines. β-actin served at the loading control.

Down-regulation of IGF-1R in ACHN cells after exposure to either Hex-hR1 or hR1 at 66 nM was apparent in 10 min, achieving a nearly complete elimination of IGF-1R at 6 h (Figure 2A). In ACHN cells, the potency of Hex-hR1 to down-regulate IGF-1R was demonstrable at 0.1 nM, as compared to that of hR1 at 1 nM (Figure 2B). At 0.1 nM, the degree to which Hex-hR1 was able to down-regulate IGF-1R was significantly higher than that of the parental hR1 (Figure 2C; *P* = 0.031).

### Surface IGF-1R expression, formation of IGF-1R/IR hybrid, and sensitivity to anti-IGF-1R treatment

IGF-1R expression levels on the cell surface were determined across a panel of eight different human RCC cell lines by hR1 staining *via* FACS analysis (Table 1). All cell lines tested were moderately to weakly positive for hR1 binding, with a range of reactivity from the highest for Caki-2 to the lowest for A-704. When compared to EGFR expression, in all cases the surface expression level of EGFR was much higher than IGF-1R in a given cell line.

**Table 1:** Surface expression of IGF-1R and EGFR as determined by flow cytometry

**Antibody**	**Median fluorescence (%Positive)**							
	**Caki-2**	**ACHN**	**CAL-54**	**786-O**	**Caki-1**	**769-P**	**A-704**	**A-498**
**FITC GAH**	**3** (**2**)	**3 (2)**	**5 (5)**	**4 (6)**	**4 (4)**	**3 (4)**	**7 (2)**	**3 (4)**
hA20*	**3 (2)**	**3 (2)**	**4 (6)**	**4 (6)**	**4 (4)**	**3 (4)**	**7 (2)**	**3 (4)**
**hR1**	**13 (70)**	**12 (52)**	**10 (36)**	**9 (71)**	**8 (21)**	**8 (21)**	**10 (5)**	**5 (21)**
h225**	**26 (95)**	**109 (99)**	**93 (99)**	**73 (98)**	**22 (76)**	**79 (98)**	**97 (92)**	**100 (98)**

*Humanized anti-CD20 antibody, veltuzumab.

**Humanized anti-EGFR antibody.

Heterodimerization of IGF-1R and insulin receptor (IR) has been linked to sensitivity to anti-IGF-1R antibodies [22]. To determine if such hybrid receptors are generally formed in RCC, all eight RCC cell lines were analyzed for the presence of IGF-1R/IR heterodimers (Figure 2D). Five of the eight cell lines demonstrated little or no presence of hybrid formation. Of these five, one (A498) had little expression of IGF-1R, two (ACHN and 786–0) had no detectable levels of IR, and the remaining two (Caki-2 and 769-P) expressed both receptors but did not show hybrid formation. Of the eight cell lines tested only three, A-704, Caki-1 and CAL-54, demonstrated the presence of IGF-1R/IR heterodimers, suggesting that IR may not play a key role in IGF-1-mediated growth stimulation of RCC.

**Figure 3:**
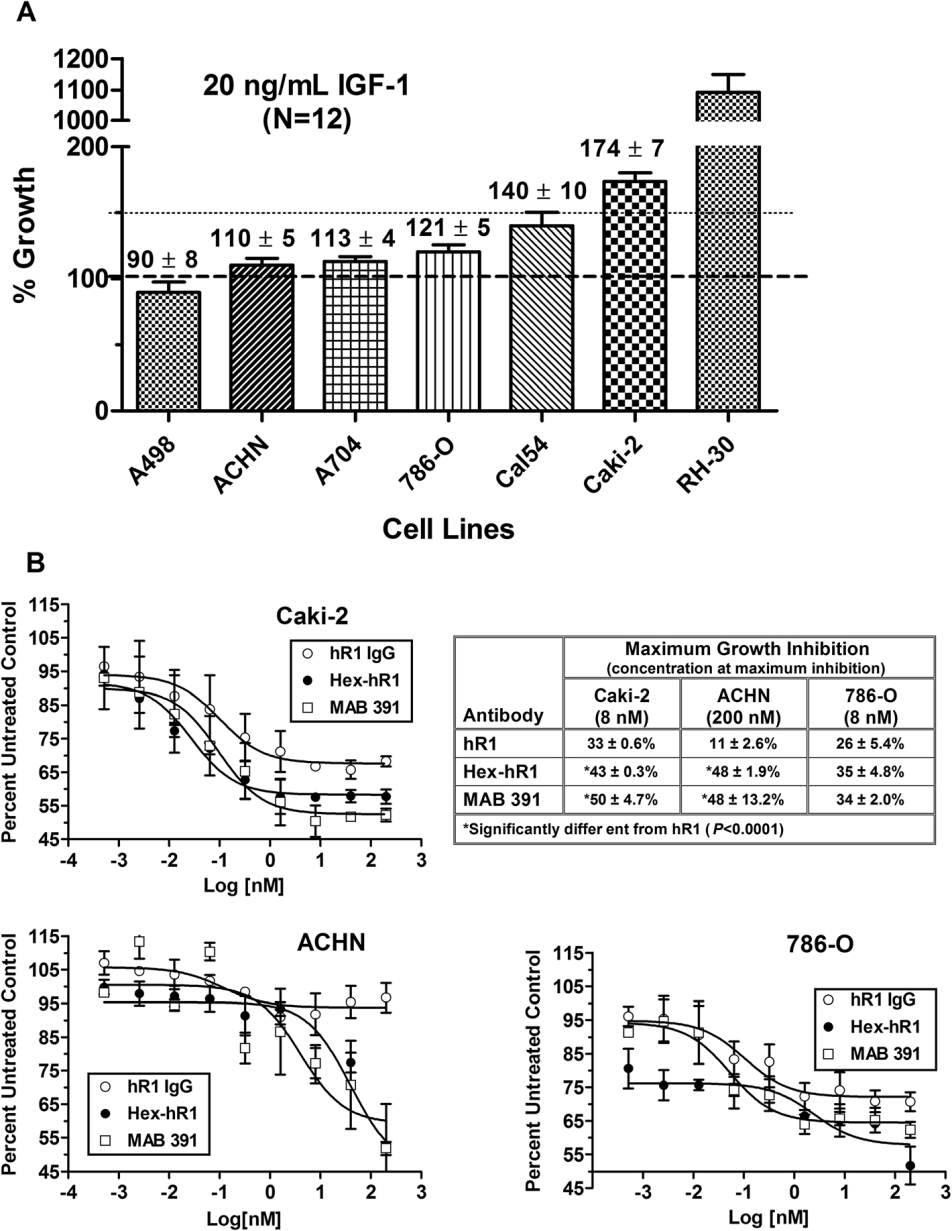
***In vitro *sensitivity to anti-IGF-1R treatment. **(**A**) Various RCC cell lines were plated in 96-well plates overnight in SFM media before being stimulate with 20 ng/mL of IGF-1. Cells were incubated a further 96 h before cell viability determined as described in Materials and Methods. Growth relative to cells grown only in SFM are shown in the graph. Large dotted line indicates growth of cells in SFM while smaller dotted line indicates point of 50% increase in growth. RH30 served as a positive control cell line. (**B**) *In vitro *cytotoxicity assay was run as described in Materials and Methods and compared hR1, Hex-hR1, and positive control MAB391. After 96-h incubation, growth as a percent of untreated cells is shown in the graphs. The table shows maximum growth-inhibition achieved in the various cell lines with each antibody and the concentration required to achieve that inhibition. Data are shown as mean ± standard deviation.

It has also been proposed that a cell line’s growth response to IGF-1 stimulation is predictive of its sensitivity to an anti-IGF-1R antibody [23]. In order to gauge possible susceptibility of various RCC cell lines to anti-IGF-1R treatment, cells were grown in SFM-Trf supplemented with human IGF-1 (20 ng/mL). Their ability to grow relative to control cells (incubated in serum-free medium only) was measured after 4 days in culture (Figure 3A), which showed several of the cell lines had a greater than 20% increase in growth. Overall, stimulation by IGF-1 followed the expression levels of IGF-1R, in that Caki-2 was the best responder (74% stimulation) and had the highest expression, while A-498 had one of the lowest expression levels and was unresponsive.

Based on the IGF-1 stimulatory effects, three RCC lines (Caki-2, ACHN, and 786-O) were selected for further testing for anti-IGF-1R-mediated growth-inhibition (Figure 3B). Consistent with the IGF-1 stimulation results, hR1 had less of an effect on inhibiting the growth of ACHN cells (10.6 ± 2.6%) when compared to Caki-2 and 786-O cells (33.3 ± 0.6% and 25.9 ± 5.4%, respectively; *P* < 0.01 *vs.* ACHN). Conversely, Hex-hR1 could inhibit growth by greater than 35% in all three cell lines, with the greatest effect in Caki-2 (43%) and ACHN (48%). In both these cell lines, this inhibition was greater than that observed with the parental hR1 antibody (*P* < 0.0001). Interestingly, control antibody MAB391 had similar activity as the Hex-hR1 in these cell lines. It should be noted that the mechanism of action for MAB391 is to block IGF-1 binding to IGF-1R [24], while Hex-hR1 down-regulates the receptor, suggesting that in these cell lines, down-regulation of the receptor with Hex-hR1 was as effective as blocking IGF-1 from binding to the receptor in inhibiting cell growth.

### *In vitro* potency of 1R-2b

**Figure 4:**
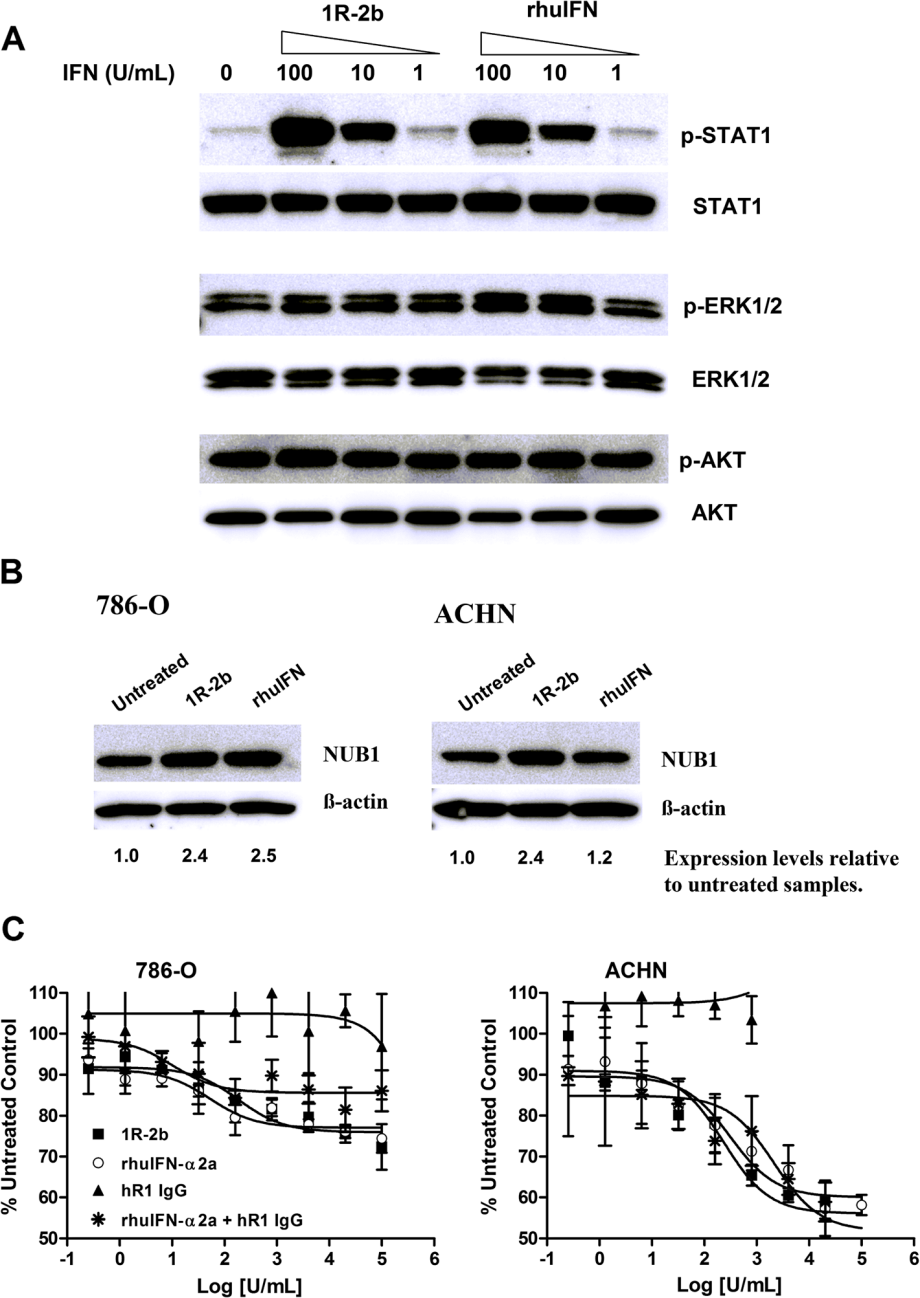
***In vitro *potency of 1R-2b. **(**A**) IFN-α mediation of phosphorylation of STAT1, ERK1/2 and AKT in ACHN cells was performed as described in Materials and Methods. Cells were exposed to the indicated amounts of 1R-2b or rhIFN-α2a for 30 min (p-STAT1) or 60 min (p-ERK1/2 and p-AKT). Fold-increase in phosphorylation was calculated relative to total protein loading controls and normalized to untreated cells. (**B**) NUB1 expression was determined as described in Materials and Methods. ACHN or 786-O cells were exposed to 3000 U/mL of 1R-2b or rhIFN-α2a for 24 h. Up-regulation was determined relative to β-actin loading control and normalized against untreated cells. (**C**) Growth inhibition was performed as described in Materials and Methods in complete media containing 10% FBS. A dose/response curve was generated with 1R-2b or rhIFN-α2a ranging from 1×10^5 ^to 0.26 U/mL. Graphs show growth relative to untreated control and represent the mean ± standard deviation.

Based on the luciferase reporter gene assay, the specific activity of 1R-2b was measured at 3750 U/pmol, which was considerably higher than peginterferon alfa-2a (180 U/pmol) and comparable to peginterferon alfa-2b (3255 U/pmol). These results are consistent with findings of other MAb-IFN agents made with the DNL methodology [19]. A further confirmation of activity was demonstrated by its ability to mediate phosphorylation of STAT1, ERK1/2 and AKT in ACHN cells (Figure 4A). When normalized to untreated control levels, both 1R-2b and rhIFN-α2a mediated a greater than 65-fold increase in p-STAT1 levels at the highest dose examined (100 U/mL). This increase in p-STAT1 levels was dose-dependent for both agents. At the intermediate doses of 10 and 1 U/mL, p-STAT1 levels were approximately 20- and 2-fold greater than control levels, respectively. The actual protein concentrations for 1R-2b and rhIFN-α2a to achieve STAT1 phosphorylation were found to be similar. For example, at 10 U/mL the amounts of 1R-2b and rhIFN-α2a were 2.7 and 2.4 pM, respectively. While both ERK1/2 and AKT were constitutively phosphorylated in untreated cells, 1R-2b mediated an approximate 2-fold increase in p-ERK1/2 and p-AKT levels at the highest dose tested of 100 U/mL, which was similar to the effects mediated by rhIFN-α2a.

Another important molecule linked to the growth-inhibitory effects of IFN in RCC is NUB1 [25]. This molecule was shown to be up-regulated by IFN in RCC cell lines, which in turn had a positive correlation with growth inhibition. The two cell lines reported to show the greatest up-regulation were ACHN and 786-O, and therefore these two were chosen to determine the effect of 1R-2b on NUB1expression (Figure 4B). Similar to what was reported with rhIFN in these two cell lines, 786-O demonstrated a greater than 2-fold up-regulation of NUB1, while ACHN had only a modest 1.2-fold increase. Likewise, when 786-O cells were incubated with 1R-2b, there was a greater than 2-fold increase in NUB1 expression that was similar to the up-regulation mediated by rhIFN-α2a. Interestingly, exposure of ACHN to 1R-2b resulted in a greater than 2-fold increase in expression, suggesting that 1R-2b may have a greater growth-inhibitory effect in ACHN than one might expect for rhIFN-α2a.

Growth-inhibitory effects of 1R-2b were examined in ACHN and 786-O cells cultured in medium containing 10% FBS (Figure 4C). In both cells, potent EC_50_ values in the picomolar range were observed for 1R-2b (63 and 48 pM in ACHN and 786-O, respectively), which were largely comparable to those of rhIFN-α2a (75 and 13 pM in ACHN and 786-O, respectively). At the maximum concentration tested (26 nM, 100,000 U/mL), 1R-2b inhibited cell growth in ACHN cells by 50.2 ± 0.5%, which was significantly better than that achieved with rhIFN-α2a at 41.9 ± 2.5% for (*P* < 0.005). There were no significant differences noted in the 786-O cells (27.6 ± 5.6% *vs.* 25.6 ± 3.5%, respectively). These data correlate with the 1R-2b-mediated up-regulation of NUB1 expression relative to rhIFN-α2a, in that 1R-2b had a greater inhibitory effect in ACHN relative to rhIFN-α2a, whereas there was no difference in 786-O.

### Synergistic Interactions of hR1, Hex-hR1, and 1R-2b with an mTOR Inhibitor

**Figure 5:**
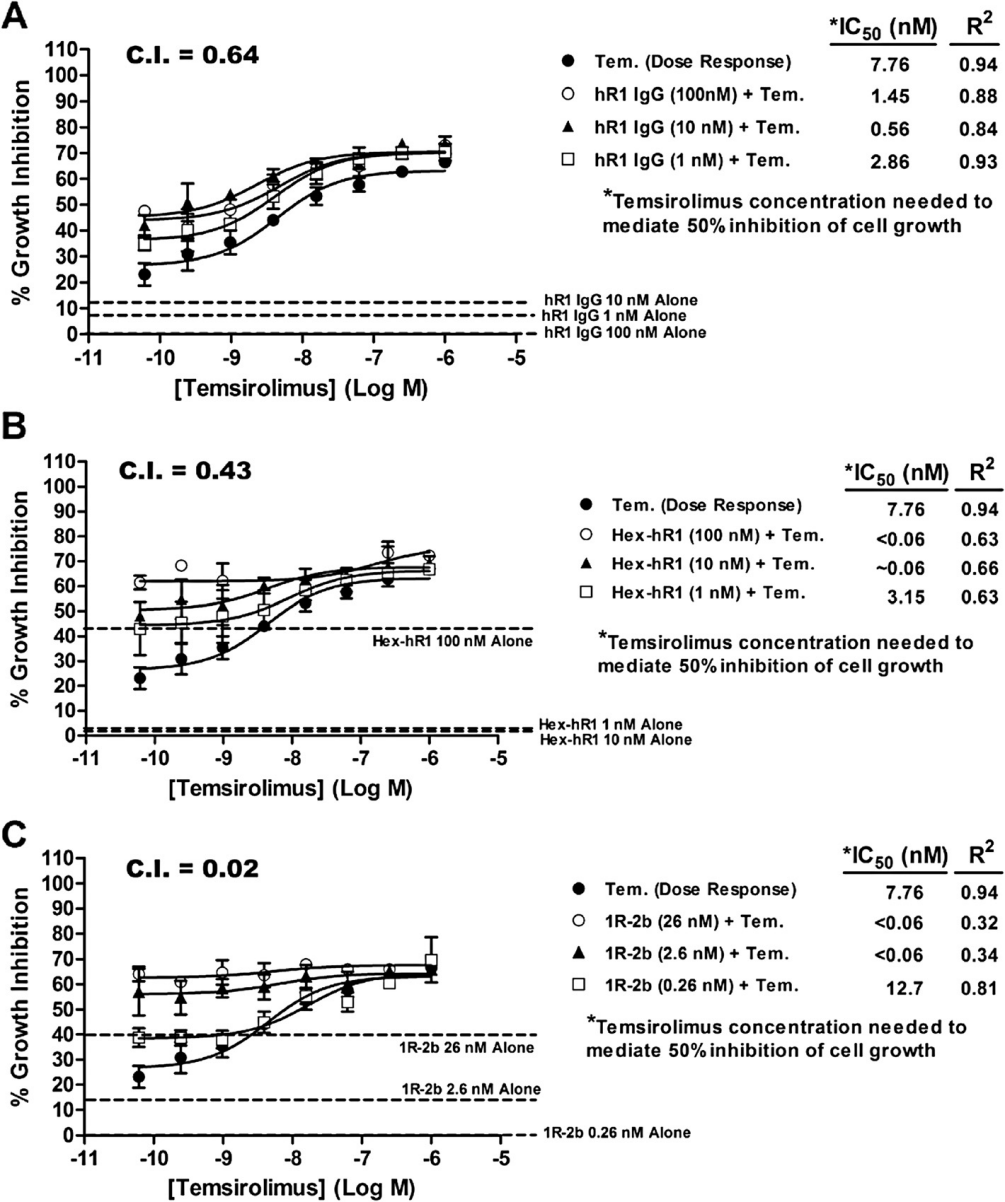
**Synergistic interaction of temsirolimus with various anti-IGF-1R molecules.** Cytotoxicity assays were performed as described in Materials and Methods. A dose/response curve with temsirolimus was made from 1x10^-6 ^to 6.1x10^-11^ M. To one set of temsirolimus wells was added a constant amount of (**A**) hR1 or (**B**) Hex-hR1 at 100 nM, 10 nM, or 1 nM. (**C**) For 1R-2b, a constant amount of 26, 2.6, or 0.26 nM was added to the wells. After 96 h incubation, growth relative to untreated cells was determined and the amount of temsirolimus required to inhibit cell growth by 50% was calculated for temsirolimus alone or when used in combination with hR1, Hex-hR1, or 1R-2b. C.I. values were determined as described, with a value less than 1 indicative of synergy. Dotted lines indicate growth-inhibition of each anti-IGF-1R agent alone. Data are shown as mean ± standard deviation.

Given the known link between signaling events mediated by IGF-1R and the mTOR pathway, the growth-inhibitory effects of hR1, Hex-hR1 and 1R-2b, when combined with the mTOR inhibitor, temsirolimus, were examined *in vitro* using ACHN as the target cell line (Figure 5). Based on a dose–response curve, the IC_50_ of temsirolimus in ACHN was 7.76 nM, which dropped to below 2.9 nM when combined with various concentrations of hR1 (100, 10, or 1 nM), indicating synergy (CI = 0.64). An even greater synergistic effect (CI = 0.43) was observed when Hex-hR1 was combined with temsirolimus (Figure 5B). At the two highest concentrations (100 and 10 nM), Hex-hR1 improved the IC_50_ by >130-fold to less than 0.06 nM. As an example of this combined effect, Hex-hR1 at 10 nM inhibited cell growth by 1.8 ± 6.2% and temsirolimus at its lowest concentration of 0.06 nM by 23.2 ± 4.3%. However, when the two were incubated together, cell growth was inhibited by 48.1 ± 1.2%. (*P* < 0.0007 *versus* either agent alone).

Since 1R-2b is effective at levels lower than hR1 or Hex-hR1, cells were incubated at concentrations of 26, 2.6, or 0.26 nM (Figure 5C). These concentrations are equivalent to 100,000 to 1000 U/mL of IFN activity. Of all three agents tested, 1R-2b had the greatest synergistic effect when combined with temsirolimus (C.I. = 0.02). At the two higher doses of 26 and 2.6 nM, the IC_50_ for temsirolimus improved to less than 0.06 nM. As an indication of this interaction, 1R-2b alone at 2.6 nM (10,000 U/mL) inhibited cell growth by 14.0 ± 8.7%; when combined with 0.06 nM temsirolimus (23.2 ± 4.3%), this improved to 56.8 ± 9.3% (*P* < 0.005 *versus* either agent alone). Overall, both Hex-hR1 and 1R-2b had a greater effect when combined with temsirolimus than the parental hR1 antibody, but all three demonstrated synergy when used in concert with this mTOR inhibitor.

## Discussion

Among kidney cancer types, approximately 90% are RCC, in which clear-cell-RCC comprises approximately 75% of all cases, and papillary RCC makes up an additional 15%. Patients present with metastatic disease 30% of the time. Unfortunately, patients with metastatic RCC have a poor prognosis, since it has remained resistant to both radiotherapy and chemotherapy [14].

Current treatments for metastatic RCC include IFN-α [7,8,26], RTK inhibitors, for example, sorafenib, sunitinib, and temsirolimus [4,5,27], and anti-VEGF receptor antibodies, such as bevacizumab [6]. These agents have been tested alone and in combinations, with some improvement in clinical outcomes.

Another approach focuses on IGF-IR as a potential therapeutic target [11-13,15]. There is evidence of an autocrine-paracrine loop in RCC growth [12], and that the expression of IGF-IR and one of its ligands, IGF-1, has a positive association with poor survival of patients with high-grade tumors [13]. By blocking IGF-IR signaling, it was shown pre-clinically that RCC growth could be reduced significantly [11] and cell invasiveness inhibited [15]. We have demonstrated that hR1 binds to multiple tumor types, including RCC [16]. Additionally, a hexavalent form of hR1 (Hex-hR1) was created by the DNL™ -platform technology. DNL explores a pair of distinct protein domains involved in the natural association between cAMP-dependent protein kinase (PKA) and A-kinase anchoring proteins (AKAPs), which can serve as linkers for site-specific conjugation of an immunoglobulin to either two dimers of IFN-α2b or four Fab fragments of an immunoglobulin, resulting in a hexavalent antibody [17-19]. Recently, in a variety of solid tumor lines, including breast, colon, and prostate, both hR1 and Hex-hR1 were shown to cause receptor down-regulation, inhibiting cell growth and invasiveness. It was noted that Hex-hR1 was much more effective at receptor down-regulation than the parental hR1. Additionally, they could both inhibit colony formation and growth in soft-agar of two human RCC cell lines [16]. In the present study, hR1 and Hex-hR1 were likewise very effective at mediating down-regulation of IGF-1R in RCC. Also, as was noted in other solid tumor lines, Hex-hR1 was more effective than hR1 at mediating receptor down-regulation at picomolar concentrations, suggesting that it may be a more potent anti-tumor agent than its parental hR1 antibody.

In human breast cancer, of 41 different cell lines tested, only 7 were sensitive to the growth-inhibitory effects of an anti-IGF-1R antibody [23]. Two of the main factors cited as predictive for anti-IGF-1R treatment were expression of IGF-1R and growth-stimulatory effects of IGF-1. In addition, the presence of IGR-1R and IR-β heterodimers in hepatic and gastric cell lines has been linked to sensitivity to anti-IGF-1R antibodies [22]. A panel of eight different human RCC cell lines was screened by FACS with hR1 for surface expression of IGF-1R and by Western blotting for IGF-1R/IR-β hybrid receptors. While all eight expressed IGF-1R, this expression varied between the cell lines, from a high in Caki-2 to a low in A-498. Conversely, only three of the RCC lines expressed the IGF-1R/IR-β heterodimer (A704, Caki-1 and CAL-54). We found that, like the breast cancer lines, RCC lines varied in their sensitivity to IGF-1 stimulation. Except for ACHN, which had IGF-1R expression similar to Caki-2, the other cell lines tested fell in the order of the higher the IGF-1R expression, the greater the effect of IGF-1 stimulation. Expression of the IGF-1R/IR-β hybrid receptor did not correlate with increased sensitivity to IGF-1 stimulation. For example, A-704, which has the heterodimer, had a lower response to IGF-1 than did Caki-2, which did not demonstrate the presence of the hybrid receptor despite expressing both IGF-1R and IR-β. To further test whether this stimulation by IGF-1 translated to growth-inhibitory effects of an anti-IGF-1R treatment, cell lines that were very sensitive (Caki-2), moderately sensitive (786-O), and with low sensitivity (ACHN) to IGF-1 treatment were incubated with hR1 or Hex-hR1 in the presence of IGF-1. As predicted by the stimulation experiment, both Caki-2 and 786-O demonstrated greater growth-inhibition by hR1 than did ACHN. Interestingly, Caki-2 also had the lowest EGFR expression and ACHN one of the highest. Over-expression of EGFR relative to normal kidney tissue has been documented in patient RCC samples, and is thought to be associated with the transformation of normal renal tissue to malignancy [28]. Additionally, it is known that both the IGF-1R and EGFR signaling pathways overlap, and both make use of phosphatidylinositol-3 kinase (PI3-K) and growth factor receptor bound protein 2 (Grb2) signaling pathways [29]. It is possible that in cell lines with high EGFR surface expression relative to IGF-1R, there may be less reliance on IGF-1 and therefore less sensitivity to the stimulatory effects of IGF-1. This was noted in hepatocellular carcinoma lines, in which resistance to anti-IGF-1R treatment was associated with increased signaling through EGFR pathways, and that this resistance was overcome by targeting both IGF-1R and EGFR [30]. It should be noted that the rhabdomyosarcoma line, RH-30, has the same surface expression of IGF-1R and EGFR when examined by FACS (data not shown). This same cell line was extremely sensitive to IGF-1, with growth-stimulation more than 10-fold above cells grown in SFM. We have demonstrated previously that this same cell line was inhibited in cell migration assays by hR1 and could be inhibited in its growth *in vivo* by both hR1 and Hex-hR1 [16]. These data suggest that EGFR expression levels relative to IGF-1R also may be predictive of sensitivity of RCC to monotherapy with an anti-IGF-1R antibody, and that targeting both IGF-1R and EGFR would be a rational approach in RCC.

While hR1 had modest effects at inhibiting cell growth, Hex-hR1 had a greater effect on RCC, even on the resistant ACHN cell line. This could be linked to the observation that the hexavalent form of hR1 is more effective at mediating down-regulation of IGF-1R than the parental hR1. Interestingly, MAB391, whose mechanisms of action include blocking IGF-1 from binding to IGF-1R as well as down-regulation of IGF-1R [24], also was as effective as Hex-hR1 at inhibiting cell growth. However, it is noted that excess IGF-1 could reverse the IGF-1-mediated signaling events blocked by MAB391 [24].

It has been observed that in cancer cells treated with rapamycin, a negative feedback resulted in which p-AKT levels increased in the cells [9,10]. Even under serum-free conditions, when IGF-1 was added to cultures, rapamycin-induced growth-inhibition was reversed and cell proliferation resumed at untreated levels [9]. One way this feedback was prevented was to treat cells with anti-IGF-1R antibodies. When rapamycin treatment was combined with anti-IGF-1R antibodies, an additive effect was achieved [9,10]. Consistent with these findings and using a similar system with RCC, cells treated with hR1 or Hex-hR1 plus temsirolimus resulted in a synergistic growth-inhibitory effect. A higher degree of growth-inhibition was achieved with Hex-hR1 when combined with temsirolimus, in which a greater than 130-fold improvement in the IC_50_ of temsiroliumus could be attained under these conditions. Such a combination had previously been tested *in vivo* in which hR1 and Hex-hR1 could significantly inhibit the growth of a rhabdomyosarcoma when each was used with rapamycin [16]. These combinations demonstrated more efficacy than when any of the three agents were used alone, and provide the rationale for such a combination in RCC.

As noted previously, efforts have also been made to use cytokine therapy, in particular IFN-α, either alone or with other treatment modalities to improve patient outcomes [4-8]. However, one of the limitations of systemic treatment with IFN-α is the adverse events associated with the therapy [4,6-8]. We have previously used the DNL method to incorporate four molecules of IFN-α into veltuzumab, an anti-CD20 antibody currently in clinical development, and shown that the resulting 20-2b had greatly improved pharmacokinetics (PK) when compared to pegylated IFN-α. We also showed that the tumor targeting ability of 20-2b allowed for a low therapeutic dose to be administered in lymphoma models [19], suggesting a high therapeutic window for this immunocytokine. In the present study, we explored the potential of 1R-2b, a new DNL-based immunocytokine comprising hR1 IgG and four IFN-α molecules, for use against RCC. 1R-2b had a specific activity similar to peginterferon alfa-2b and superior to peginterferon alfa-2a. When compared to IFN-α, 1R-2b had the same ability to mediate phosphorylation of STAT1, AKT and ERK1/2 at comparable concentrations. Additionally, 1R-2b could inhibit the growth of two different RCC lines in a similar manner as free IFN-α, indicating that the IFN-α on 1R-2b is fully functional.

This growth-inhibitory effect by IFN-α has been demonstrated previously in several human RCC lines, including ACHN and 786-O [25]. One main mechanism of action of IFN-α described in these RCC lines is the up-regulation of the NEDD8 ultimate buster I (NUBI) protein. There is a positive correlation in NUBI up-regulation and cell growth inhibition in RCC [25]. NEDD8 is an ubiquitin-like molecule that covalently binds to several different proteins including the VHL protein (pVHL) [31]. Normally, pVHL targets proteins in the hypoxia-inducible factor (HIF)-α family for ubiquitination and subsequent destruction. Defects in this gene, as in many RCC tumors, result in the accumulation of HIF proteins which in turn activate other growth factor genes that promote angiogenesis and cell proliferation associated with RCC [32]. NEDD8 covalently binds to pVHL and modifies it to allow for the proper assembly of the fibronectin matrix, but failure to bind does not affect the ability of pVHL to ubiquinate HIF [31]. In pVHL mutants, failure of fibronectin assembly is associated with an undifferentiated phenotype in RCC [33]. RCC cells treated with IFN-α exhibit an up-regulation in cyclin E and p27 and the down-regulation of NEDD8, which results in the accumulation of cells in S-phase and induction of apoptosis. It is thought that the NEDD8 conjugation system plays a role in the ubiquitination of p27 and cyclin E such that NUBI-mediated degradation of NEDD8 induces growth arrest and apoptosis of RCC [25]. When ACHN and 786-O were exposed to 1R-2b, there was a greater than 2-fold increase in NUB1 expression. However, while rhIFN-α2a had a similar effect in 786–0, in ACHN 1R-2b mediated a higher increase in expression levels than what was observed with rhIFN-α2a. Likewise, similar to what was reported previously [25], a correlation between NUB1 expression and growth inhibition was observed with 1R-2b in ACHN, in that 1R-2b resulted in a significantly greater degree of growth inhibition when compared to rhIFN-α2a.

Inhibition of mTOR likewise affects the cell cycle, with a down-regulation of cyclin D and the inability to progress from G_1_ to S-phase [5]. There is also evidence that Type I IFNs, such as IFN-α, may have parallel AKT/mTOR signaling pathways that promote growth-inhibitory signals [34], and that by blocking mTOR it may antagonize the effect of IFN-α treatment. To determine if this action by IFN-α would have an added or antagonistic effect when combined with the mTOR inhibitor, temsirolimus, RCC cells were incubated with both temsirolimus and 1R-2b. This combination proved to be very potent, with a synergistic effect observed at a 1R-2b concentration as low as 2.6 nM. These data indicate that even if IFN-α used the mTOR pathway in parallel to inhibit cell growth in RCC, it must play only a minor role, because blocking such activity by temsirolimus did not blunt the effect of 1R-2b, and actually worked in synergy with it to inhibit cell growth. Given the improved PK profile of the MAb-2b construct in comparison to PEGylated-IFN-α [19] and its added ability to potentially target the IFN to the tumor, 1R-2b should have the ability to be active clinically at doses substantially lower than currently administered IFN-α. Moreover, this synergistic effect, when combined with temsirolimus, provides the rationale for clinical use of 1R-2b to mitigate the dose-limiting toxicity associated with systemic administration of IFN-α when used in concert with RTK inhibitors.

In summary, monotherapy with mTOR inhibitors has met with only modest effects clinically due mainly to the ability of some cancer cells to use signaling pathways upstream and parallel to mTOR to overcome this inhibition [35]. Two such upstream pathways originate with IGF-1R, which will activate Akt and Ras when bound by its ligand [36]. It has been demonstrated that depletion of RCC cells of IGF-1R with small interfering RNA (siRNA) enhanced the sensitivity of these cells to the inhibitory effects of rapamycin [37]. Here we show that this same effect in RCC can be accomplished with hR1 and Hex-hR1. We believe that by using hR1 or Hex-hR1 to down-regulate IGF-1R in combination with temsirolimus, multiple proliferation pathways are blocked, leaving RCC little chance to bypass them and escape death. In a similar fashion, 1R-2b may affect a cell’s ability to progress through a normal cell cycle by the accumulation of cyclin E and p27, resulting in arrest at the S-phase [25]. Inhibition of mTOR also will block a normal progression through the cell cycle by the down-regulation of cyclin D [5]. Again, by combining 1R-2b with temsirolimus, two different pathways that lead to normal progression through the cell cycle may be affected, resulting in cell cycle arrest and inhibition of cell growth.

## Conclusions

We have demonstrated that by targeting multiple cell proliferation pathways in RCC simultaneously, a potent growth-inhibitory effect is observed *in vitro*. In particular, by using a humanized anti-IGF-1R antibody and its hexavalent form to mediate IGF-1R down-regulation in concert with the mTOR inhibitor, temsirolimus, cell growth is effectively blocked. Additionally, using our DNL-platform to make an anti-IGF-1R/IFN-α immunocytokine, a very potent therapeutic was created that also synergizes with temsirolimus to inhibit RCC cell growth. Finally, since DNL lends itself to design many combinations of different antibodies, possibly more potent bispecific antibodies can be generated from hR1 and anti-EGFR or -VEGF antibodies to target multiple growth pathways in RCC. Given the potent activity these anti-IGF-1R agents demonstrate against RCC when combined with temsirolimus, such a combination may prove to be beneficial clinically in the management of RCC.

## Competing interests

All authors have employment and stock or stock options with Immunomedics, Inc.

## Authors’ contributions

Conceived and designed the experiments: TMC, DMG, CHC. Performed the experiments: PT, RA. Analyzed the data: TMC, CHC, PT, RA. Contributed reagents/materials: CHC. Wrote and revised the paper: TMC, DMG, CHC. Provided financial support: DMG. All authors read and approved the final manuscript.

## Pre-publication history

The pre-publication history for this paper can be accessed here:

http://www.biomedcentral.com/1471-2407/13/170/prepub
